# Identification of genes differentially expressed in a resistant reaction to *Mycosphaerella pinodes *in pea using microarray technology

**DOI:** 10.1186/1471-2164-12-28

**Published:** 2011-01-13

**Authors:** Sara Fondevilla, Helge Küster, Franziska Krajinski, José I Cubero, Diego Rubiales

**Affiliations:** 1 Department of Genetics, University of Córdoba, Campus de Rabanales, E-14071, Córdoba, Spain; 2 Institute for Plant Genetics, Unit IV - Plant Genomics, Leibniz Universität Hannover, Herrenhäuser Str. 2, D-30419 Hannover, Germany; 3 Max-Planck Institute of Molecular Plant Physiology, Wissenschaftspark Golm, Am Muehlenberg 1, 14476,Potsdam, Germany; 4 CSIC, Institute for Sustainable Agriculture, Apdo. 4084, E-14080,Córdoba, Spain

## Abstract

**Background:**

Ascochyta blight, caused by *Mycosphaerella pinodes *is one of the most important pea pathogens. However, little is known about the genes and mechanisms of resistance acting against *M. pinodes *in pea. Resistance identified so far to this pathogen is incomplete, polygenic and scarce in pea, being most common in *Pisum *relatives. The identification of the genes underlying resistance would increase our knowledge about *M. pinodes-*pea interaction and would facilitate the introgression of resistance into pea varieties. In the present study differentially expressed genes in the resistant *P. sativum *ssp. *syriacum *accession P665 comparing to the susceptible pea cv. Messire after inoculation with *M. pinodes *have been identified using a *M. truncatula *microarray.

**Results:**

Of the 16,470 sequences analysed, 346 were differentially regulated. Differentially regulated genes belonged to almost all functional categories and included genes involved in defense such as genes involved in cell wall reinforcement, phenylpropanoid and phytoalexins metabolism, pathogenesis- related (PR) proteins and detoxification processes. Genes associated with jasmonic acid (JA) and ethylene signal transduction pathways were induced suggesting that the response to *M. pinodes *in pea is regulated via JA and ET pathways. Expression levels of ten differentially regulated genes were validated in inoculated and control plants using qRT-PCR showing that the P665 accession shows constitutively an increased expression of the defense related genes as peroxidases, disease resistance response protein 39 (DRR230-b), glutathione S-transferase (GST) and 6a-hydroxymaackiain methyltransferase.

**Conclusions:**

Through this study a global view of genes expressed during resistance to *M. pinodes *has been obtained, giving relevant information about the mechanisms and pathways conferring resistance to this important disease. In addition, the *M. truncatula *microarray represents an efficient tool to identify candidate genes controlling resistance to *M. pinodes *in pea.

## Background

Legumes are a versatile and inexpensive source of protein for human food and animal feed. In addition, legumes provide numerous environmental benefits that could contribute to the sustainability of agriculture. Legumes are able to symbiotically fix atmospheric nitrogen, improving soil fertility and decreasing N fertilizers needs [1].

Dry pea is the most produced legume in Europe and the fourth most in the world [2] and one of the most productive. However, the instability of pea yields, caused mainly by the occurrence of diseases, hampers the expansion of this legume.

Ascochyta blight, caused by *Mycosphaerella pinodes *(Berk & Blox) Vesterg, the teleomorph of *Ascochyta pinodes *(Berk & Blox) Jones, is one of the most important pea pathogens [3]. It is widespread throughout the major pea growing areas [4,5], especially in temperate regions of Europe, North America, Australia and New Zealand [4] and constitutes the major constraint for the crop after broomrape in the Mediterranean basin [6]. The disease causes 10% yield losses as an average and can reach 50% under some conditions [7]. The use of resistant varieties would be the most efficient, economical and ecologically strategy to control the disease. However, pea varieties resistant to *M. pinodes *are not available.

Complete resistance to *M. pinodes *has not been identified so far. Although extensive searches have been carried out, only moderate resistance has been reported in the cultivated pea [8-10] and this has been inadequate to control the disease. Higher levels of resistance have been identified in wild species of *Pisum *[8,11,12], but their use in breeding programs is hampered by the polygenic nature of resistance.

The identification of the genes controlling resistance to *M. pinodes *in these wild resistant accessions would facilitate their introgression into pea varieties but these genes are difficult to identify by traditional approaches. Quantitative Trait Loci (QTL) analysis have identified numerous genomic regions involved in resistance to this disease in pea [13-17]. In addition, candidate genes approaches and comparative mapping have revealed the co-localization of QTLs for resistance to *M. pinodes *and resistance gene analogs, the putative transcription factor *PsDof1 *and the pea defensin DRR230-b [18,16], but still very little is known about the mechanisms of response to *M. pinodes *in pea at the histological, molecular and biochemical level.

Large scale expression studies would allow the establishment of a global and detailed picture of all genes and metabolic pathways expressed or differentially regulated during *M. pinodes*-pea interaction and would contribute to the identification of candidate genes implicated in ascochyta blight resistance. However, this approach has never been performed in this pathosystem.

The goal of this study was to identify genes and mechanisms of resistance underlying phenotypic variation in resistance to *M. pinodes *in pea using microarray technology. The advent of microarray technology has enabled large-scale surveys leading to a more integrated view of gene expression responses [19]. In plant-pathogen interactions microarray studies allow a more comprehensive understanding of molecular responses in the infection process making the elucidation of mechanisms involved in resistance possible [20]. The microarray technology requires prior knowledge of the sequence of the genome, but sequence information of pea is at the moment limited. Therefore, this study has taken advantage of the knowledge and tools developed in the model legume *Medicago truncatula*. A microarray (Mt16KOLI1Plus) [21] containing 16,470 different 70 mer oligonucleotides from *M. truncatula*, that represent all tentative consensus sequences (TCs) of the TIGR *M. truncatula *Gene Index 5 (http://compbio.dfci.harvard.edu/tgi/cgi-bin/tgi/gimain.pl?gudb=medicago) is available. In the present study cDNA obtained from resistant and susceptible pea plants inoculated with *M. pinodes *has been hybridised to this microarray and genes differentially expressed in the resistant genotype during *M. pinodes *infection have been identified.

## Results

### Microarray experiment

**Figure 1:**
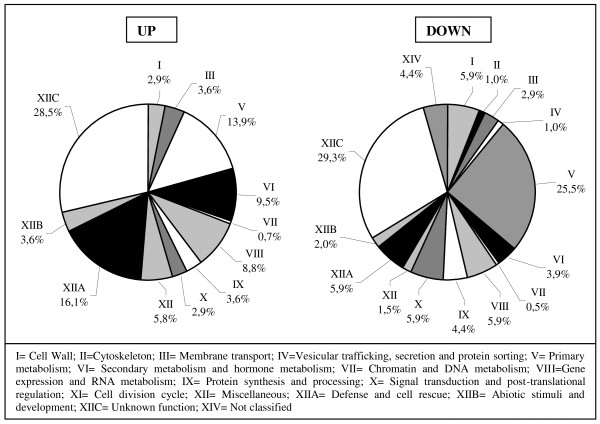
**Functional classification of genes differentially regulated in P665, comparing to Messire, after inoculation with *M. pinodes***. Functional classification according to Journet et al. [22]) of genes up or down regulated in accession P665, comparing to Messire, after inoculation with *M. pinodes*. A gene was considered to be up/down regulated when M ≥ 0.8/M ≤ -0.8 and p ≤ 0.05 in at least one time point.

Of the 16,470 sequences included in the microarray, only 25 did not show an analizable signal in any of the time points studied and the vast majority of them showed an analyzable signal in all the time points included. Of the sequences analysed, 346 were significantly differentially regulated in P665 compared to Messire in at least one time point (M ≥ 0.8 or M ≤ -0.8, p ≤ 0.05). A complete list of these genes is included in Additional file 1, Table S1. Of them, around 70% showed sequences similarities to existing sequence entries of known function in the databases. The remaining (30%) represented sequences of currently unknown functions (Figure 1). Genes differentially regulated belonged to almost all functional categories described by Journet et al. [22]. In the case of genes with higher transcript levels in P665 than in Messire (called up-regulated in this paper), the largest proportion belonged to the category 'Defense and cell rescue' (16,1%), followed by 'Primary metabolism' (13.9%). Genes included in the categories 'Secondary metabolism and hormone metabolism' (9.5%) and 'Gene expression and RNA metabolism' (8.8%) were also abundant. Categories 'Miscellaneous' (5.8%), 'Membrane transport' (3.6%), 'Cell Wall', 'Protein synthesis and processing', and 'Signal transduction and post-translational regulation' (around 2.9% each) were also present. Only one gene of the category 'Chromatin and DNA metabolism' was up regulated and none in the categories 'Cytoskeleton' and 'Vesicular trafficking, secretion and protein sorting'.

In the case of genes less expressed in P665 than in Messire (called down-regulated), the most abundant category was 'Primary metabolism' (25.5%). Categories **'**Signal transduction and post-translational regulation', 'Gene expression and RNA metabolism', 'Defense and cell rescue' and 'Cell Wall' formed a second group representing around 6% each. The percentage of genes included in the categories 'Protein synthesis and processing', 'Secondary metabolism and hormone metabolism', 'Membrane transport' or not classified in any category, ranged from 2.9 to 4.4 while the remaining categories were weakly represented.

**Figure 2:**
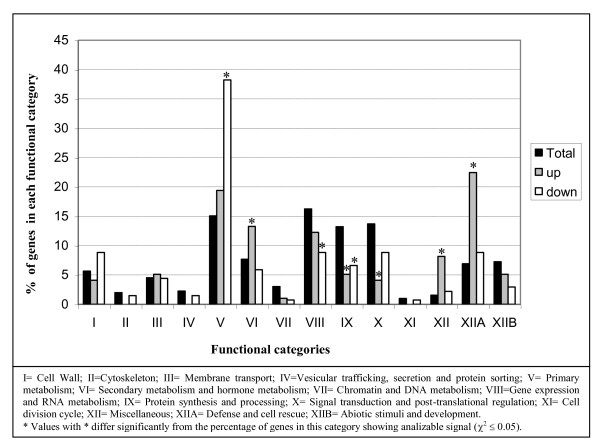
**Functional classification of genes showing analysable signal compared to up/down regulated genes**. Percentage of genes in each functional category for which there was analyzable signal on the array (Total) compared to the percentage of up/down regulated genes in each category. Genes with unknown function or that could not be classified in any category were not included in the analysis.

The up regulation of genes belonging to the functional categories 'Defense and cell rescue' and 'Secondary metabolism and hormone metabolism' and the down regulation of genes involved in 'Primary metabolism' in P665 infected plants comparing to Messire was confirmed by the statistically significant higher percentage of differentially expressed genes included in these categories compared to the percentage of genes in these functional categories for which there was analyzable signal on the array (Figure 2). The category miscellaneus also showed some grade of up regulation. In contrast, a certain depletion was identified in the set of up regulated genes for the categories 'Protein synthesis and processing' and 'Signal transduction and post-translational regulation' and in the set of down regulated genes for the categories 'Gene expression and RNA metabolism' and' Protein synthesis and processing'.

The genes differentially regulated in P665 comparing to Messire belonging to the categories: 'Cell wall', 'Secondary metabolism and hormone metabolism', 'Gene expression and RNA metabolism', 'Signal transduction and post-translational regulation', 'Defense and cell rescue' and 'Abiotic stimuli and development' are shown in Table 1.

**Table 1:** Most relevant genes differentially expressed in P665 compared to Messire after inoculation with *M. pinodes*

**Oligo ID**^**a**^	**TIGR ID**^**b**^	Annotation	**M16**^**c**^	M24	M48	FC
MT015143	BG584806	Repetitive proline-rich cell wall protein 2 precursor	1.39*	1.04*	1.30*	I
MT015121	BG586912	Repetitive proline-rich cell wall protein 1 precursor	1.22*	0.69	1.05*	I
MT002297	TC79657	Nodulin-like protein	1.19*	0.86*	0.86*	I
MT005666	TC83381	Caffeic acid O-methyltransferase	0.17	0.22	0.88*	I
MT004103	TC82015	Beta-galactosidase	0.03	-0.08	-1.13*	I
MT014461	TC86053	Beta-galactosidase	-0.18	-0.40	-1.23*	I
MT013510	TC91374	Probable xyloglucan endotransglycosylase	-0.44	-1.08*	-0.61	I
MT006437	TC77501	Endoxyloglucan transferase	-0.71	0.12	-1.42	I
MT014283	TC85611	Caffeoyl-CoA O-methyltransferase	-0.87*	-0.12	-1.55*	I
MT014301	TC76880	Endoxyloglucan transferase	-0.90*	0.08	-0.51	I
MT014287	TC76828	Extensin-like protein	-1.00*	0.12	-0.43	I
MT001416	TC78670	Expansin	-1.01*	0.31	0.19	I
MT014300	BQ136812	Xyloglucan endotransglycosylase	-1.05*	0.15	-0.82*	I
MT015434	TC86491	Expansin	-1.09*	-0.44	-1.21*	I
MT009043	TC78936	Protein T10O24.17	-1.14*	-1.38*	-1.06*	I
MT007149	TC76727	Putative proline-rich protein APG isolog	-1.49*	-0.81*	-0.45	I
MT015061	TC85201	Lipoxygenase	1.42*	0.88*	0.44	VI
MT013322	TC93346	Peroxisomal copper-containing amine oxidase	0.99*	0.27	0.02	VI
MT006064	TC91378	AT3g62110	0.95*	0.85*	0.67	VI
MT000596	TC86308	Cytochrome P450 78A3	0.74	-0.71	-1.90*	VI
MT005593	TC83534	Putative amine oxidase	0.57	-0.14	1.46*	VI
MT001070	TC78077	UDP-glycose flavonoid glycosyltransferase	0.57	0.85*	0.20	VI
MT006134	TC81027	4-coumarate--CoA ligase-like protein	0.38	1.37*	0.1	VI
MT006994	TC85176	Lipoxygenase	0.32	0.25	-1.54*	VI
MT000200	TC85619	Probable lipoxygenase	0.17	0.07	0.86*	VI
MT000193	TC85559	Acetyl-CoA carboxylase	0.13	1.34*	0.06	VI
MT000333	TC85502	Phenylalanine ammonia-lyase	0.06	1.32*	-0.44	VI
MT013671	TC84229	Cytochrome p450	0.06	-0.66	-0.87*	VI
MT012159	TC83702	Squalene monooxygenase	0.05	0.94*	-0.47	VI
MT003115	TC89052	Cytochrome P-450LXXIA1 (cyp71A1)	0.05	0.21	1.23*	VI
MT001043	TC77410	Flavanone 3 beta-hydroxylase	0.02	0.15	1.49*	VI
MT002497	TC88443	Hyoscyamine 6 beta-hydroxylase	-0.13	0.53	0.96*	VI
MT009587	TC78460	Flavonol synthase-like protein	-0.81*	-0.42	-0.47	VI
MT003010	TC89135	Cytochrome P450 71A21	-1.06*	-2.15*	-0.37	VI
MT014118	TC85168	Lipoxygenase	-1.48*	-1.73*	0.24	VI
MT002785	TC80364	Tropinone reductase homolog	-1.32*	-1.40*	-1.53*	VI
MT012682	TC80051	Amygdalin hydrolase isoform AH I precursor	-1.39*	-0.48	-0.45	VI
MT001339	TC87447	NAC domain protein NAC1	1.26*	1.11*	0.28	VIII
MT009598	TC79845	Ethylene responsive element binding factor-like	1.09*	1.43*	0.39	VIII
MT008649	TC87796	EBNA-1 nuclear protein	1.06*	1.00*	0.60	VIII
MT005976	TC93710	Promoter-binding factor-like protein	1.03*	0.91*	0.80	VIII
MT000362	TC77110	Putative steroid membrane binding protein	1.01*	1.01*	1.17*	VIII
MT016167	BI262875	GRAS family transcription factor	0.90*	0.66	0.13	VIII
MT015261	TC76796	Transcription factor JERF1	0.89*	0.97*	0.29	VIII
MT016157	BE319790	Pathogenesis related transcriptional factor and ERF	0.88*	0.02	-0.29	VIII
MT002827	TC80713	Probable cysteinyl-tRNA synthetase	0.86*	0.83*	0.38	VIII
MT008661	TC87393	Probable CCCH-type zinc finger protein	0.85*	0.24	-0.27	VIII
MT016437	AJ848040	Probable C2H2 type zinc finger protein ID1 like	0.33	0.86*	-0.13	VIII
MT011589	TC87048	F5M15.3	-0.07	-1.13*	-1.98*	VIII
MT008378	TC87360	Chromosome chr7 scaffold_31 whole genome shotgun sequence	-0.19	-0.15	-0.83*	VIII
MT008731	TC78273	Putative transcription factor APFI	-0.31	-0.88*	-0.13	VIII
MT015990	TC76565	AT3g16857	-0.40	-1.41*	-0.50	VIII
MT013325	TC84058	GATA-binding transcription factor-like protein	-0.52	-0.39	-1.09*	VIII
MT009202	TC77895	AT4g00150	-0.58	0.48	1.51*	VIII
MT011704	TC91195	Putative CTP synthase	-0.81*	-0.81*	-0.66*	VIII
MT002341	TC79766	bHLH transcription factor GBOF-1	-0.84*	-0.13	0.18	VIII
MT011401	TC79757	AT3g09731	-1.03*	-0.61	-0.59	VIII
MT015283	TC85653	SRG1 protein	-1.12*	-0.05	0.15	VIII
MT015124	AL371197	Glycine-rich RNA binding protein	-1.30*	-0.61	-0.28	VIII
MT011927	TC90604	Probable homeobox protein T9L24.43	-1.47	-0.11	0.22	VIII
MT012751	TC83617	Transcriptional regulator AraC family	-1.69*	-0.94*	-1.32	VIII
MT014356	TC85808	12-oxophytodienoate reductase (OPR2)	1.27*	1.06*	0.03	X
MT014354	TC85808	12-oxophytodienoate reductase (OPR2)	1.19*	0.71	-0.25	X
MT011343	TC80935	Receptor protein-like	1.15*	0.88*	0.03	X
MT011648	TC91194	Putative wall associated serine/theorine kinase	0.81*	-0.01	0.39	X
MT002252	TC87949	Calcium/calmodulin-dependent protein kinase CaMK2	0.06	-0.20	-0.91*	X
MT002729	TC89621	At2g23450	0.04	-0.18	-1.07*	X
MT009158	TC88304	F12A21.14	-0.10	-1.28*	-0.40	X
MT014753	TC90240	Ser/Thr kinase	-0.15	0.48	-1.38*	X
MT013890	TC88105	Putative protein kinase	-0.23	-0.17	-0.98*	X
MT008763	TC79044	Protein kinase	-0.40	0.44	-1.00*	X
MT010193	TC80761	RAB1Y	-0.73	-2.25*	-0.76	X
MT015663	TC88029	Signal peptidase	-0.83*	-0.80	-0.47	X
MT008494	TC78698	Signal recognition particle 54 kDa subunit precursor	-0.88*	-0.58	-1.13*	X
MT009940	BI263421	Mitogen-activated protein kinase	-1.39*	-0.87*	-1.12*	X
MT012718	TC83265	Guanylate kinase	-1.85*	-0.22	-0.29	X
MT010223	TC90306	At1g21410	-1.86*	0.13	0.77	X
MT014704	TC80412	Peroxidase	3.61*	3.08*	1.56*	XIIA
MT001261	TC87286	Nine-cis-epoxycarotenoid dioxygenase4	2.06*	1.68*	-0.05	XIIA
MT015019	TC93000	Bacterial-induced peroxidase precursor	1.97*	1.32*	1.02*	XIIA
MT014726	TC79559	Glutathione S-transferase	1.96*	2.08*	-	XIIA
MT008551	TC78940	Cationic peroxidase 2 precursor	1.77*	1.20*	-0.28	XIIA
MT012181	C89099	Lipid transfer protein SDi-9 drought-induced	1.60*	1.20*	1.23*	XIIA
MT008366	TC78224	Bacterial-induced peroxidase precursor	1.50*	1.77*	0.98*	XIIA
MT000911	TC76930	Syringolide-induced protein B13-1-9	1.38*	1.13*	1.00*	XIIA
MT006316	TC82368	Disease resistance response protein 39 precursor	1.29*	1.49*	1.13*	XIIA
MT006497	TC93816	ABC transporter	1.29*	1.24*	1.10*	XIIA
MT006999	TC85204	Peroxidase1A	1.28*	1.60*	1.81*	XIIA
MT007682	TC77455	Nine-cis-epoxycarotenoid dioxygenase1	1.12*	1.09*	0.88*	XIIA
MT015903	TC82203	Peroxidase	1.01*	0.76	0.39	XIIA
MT015524	TC77937	CjMDR1	0.98*	0.61	0.15	XIIA
MT014728	TC78224	Bacterial-induced peroxidase precursor	0.91*	0.69	0.33	XIIA
MT015980	TC82138	Probable glutathione S-transferase	0.86*	0.71	0.36	XIIA
MT009791	TC86798	Cyanogenic Beta-Glucosidase Molid 1	0.80*	0.14	-0.24	XIIA
MT015051	TC85153	Peroxidase precursor	0.48	0.98*	1.11*	XIIA
MT015067	TC85170	Peroxidase	0.37	0.85*	1.17*	XIIA
MT014072	TC77400	Beta-1 3-glucanase	0.13	0.41	-1.35*	XIIA
MT007613	TC86304	GA protein	0.10	0.10	-2.28*	XIIA
MT014080	TC85172	Peroxidase 3	0.04	0.96*	0.01	XIIA
MT015763	TC81227	Elicitor inducible gene product Nt-SubE80	0.03	-0.16	-0.87*	XIIA
MT015058	TC85182	Peroxidase	-0.35	0.65	-1.17*	XIIA
MT006425	MT006425	Disease resistance protein-like	-0.39	-0.01	0.89*	XIIA
MT014328	TC85843	5-epi-aristolochene synthase	-0.44	-0.11	0.81*	XIIA
MT014169	TC76642	Pprg2 protein	-0.72	-1.04*	-0.38	XIIA
MT008899	TC79452	TIR-similar-domain-containing protein TSDC	-0.79	-1.10*	-1.62*	XIIA
MT003218	TC90010	Putative NBS-LRR type disease resistance protein	-0.84*	-0.65	-0.94*	XIIA
MT000053	BM813626	Ascorbate peroxidase	-0.90*	-0.78	-0.42	XIIA
MT011658	TC82236	Putative resistance protein	-1.18*	-0.68	-3.38*	XIIA
MT015567	TC78525	Syringolide-induced protein 19-1-5	-1.32*	-0.81*	-0.39	XIIA
MT000707	TC86358	6a-hydroxymaackiain methyltransferase	-2.13*	-1.98*	-1.71*	XIIA
MT015446	TC77584	Epoxide hydrolase homolog	-3.48	-0.40	-1.21*	XIIA
MT015286	TC85739	Ripening-related protein-like	1.66*	0.84*	-0.16	XIIB
MT015373	TC85963	CIC protein cold-inducible	1.65*	0.98*	1.00*	XIIB
MT003152	TC88482	Auxin influx carrier protein	1.50*	1.19*	1.17*	XIIB
MT009872	TC80360	Probable wound-induced protein T9A4.6	0.94*	0.62	-0.09	XIIB
MT012817	TC91709	LHY protein	0.09	0.81*	-0.70	XIIB
MT001051	TC78061	Auxin-induced protein	0.68	0.63	-0.87*	XIIB
MT013626	TC82806	GMFP7	-0.70	-0.43	-0.83*	XIIB
MT014513	TC78341	Embryo-specific protein-like	-1.08*	-0.10	-0.54	XIIB
MT001024	TC79562	Putative 16.9 kDa heat shock protein	-1.12*	-0.22	-1.04*	XIIB

^a ^Oligo ID, identifier of *M. truncatula *70-mer oligonucleotides

^b ^TIGR ID, identifier in the TIGR *M. truncatula *Gene Index;

^c ^M = log_2 _expression ratio P665/Messire at 16, 24 and 48 hours after inoculation

* M ≤ -0.8 or M ≥ 0.8 are significant at significance level of 0.05 using t-test and FDR correction

Genes of the functional categories (FC): I (Cell wall), VI (Secondary metabolism and hormone metabolism), VIII (Gene expression and RNA metabolism), X (Signal transduction and post-translational regulation), XIIA (Defense and cell rescue) and XIIB (Abiotic stimuli and development) differentially expressed in P665 compared to Messire after inoculation with *M. pinodes*.

#### Cell wall

Sixteen sequences involved in cell wall were differentially expressed in inoculated P665 plants comparing to Messire. Genes implicated in cell wall reinforcement were in general more expressed in P665 than in Messire while genes involved in cell elongation, wall expansion and wall degradation were less expressed.

#### Membrane transport

Up-regulated genes belonging to this category included those associated with protein and amino acid transport and a putative Na+/H+ antiporter. In contrast, several putative membrane transporter proteins and a gene involved in potassium transport were down regulated.

#### Primary metabolism

A high percentage of genes differentially expressed in P665 comparing to Messire corresponded to genes involved in primary metabolism. Most of them (73%) showed down regulation. Of them, numerous sequences corresponded to genes participating in photosynthesis. Other down-regulated genes of this category were involved in mobilization and degradation of carbohydrates, degradation of storage oil and nitrogen metabolism. Interestingly, two sequences encoding NADH-plastoquinone oxidoreductase chain 1 chloroplast were also down regulated.

Genes up-regulated included glucosyltransferases, a probable anthocyanin 5-aromatic acyltransferase, a putative acid phosphatase and genes involved in amino acid and phosphor metabolism.

#### Secondary metabolism and hormone metabolism

The secondary metabolism plays an important role in the response against pathogens. Several genes associated with the synthesis of antimicrobial compounds and involved in defense were more expressed in P665 compared to Messire after inoculation with *M. pinodes*. Among them, genes involved in phenylpropanoid, alkaloid and flavonoid metabolism, a sequence encoding phenylalanine ammonia-lyase, 2 sequences encoding lipoxygenases and genes involved in H_2_O_2 _production were included. However, a sequence showing similarities with a lipoxygenase, another encoding tropinone reductase homolog and a flavonol synthase-like protein were down regulated.

#### Gene expression and RNA metabolism

Several transcription factors and binding proteins were differentially regulated in P665 comparing to Messire. Genes up regulated encoded a NAC domain protein, an ethylene responsive element binding factor and transcriptions factors belonging to ERF and GRAS families. Transcription factors belonging to bHLH and GATA family were down regulated.

#### Signal transduction and post-translational regulation

Interestingly, two sequences encoding a 12-oxophytodienoate reductase (OPR2) were more expressed in P665 than in Messire. Several protein kinases involved in different processes were less expressed.

#### Miscellaneous

Eleven sequences included in the 'Miscellaneous' category, according to Journet et al. [22] showed a different regulation in P665 compared to Messire. Among them there were three up-regulated sequences encoding lectins, proteins that can be involved in defense. Other up-regulated genes encoded a beta-glucosidase and an early light inducible protein. A CLC-b chloride channel protein was down regulated. Genes similar to dermal glycoproteins and a legumin were represented by different sequences that were up-regulated in some cases and down regulated in others.

#### Defense and cell rescue

As expected, numerous sequences corresponding to genes involved in defense were more expressed in P665 comparing to Messire. Those included, among others, sequences encoding peroxidases, a disease resistance response protein 39 precursor, nine-cis-epoxycarotenoid dioxygenase and glutathione S-transferases. However other proteins with possible roles in defense were less expressed in P665. Those included GA protein, PR-10, ascorbate peroxidase and a putative NBS-LRR type disease resistance protein.

#### Abiotic stimuli and development

Different proteins related to the response to 'Abiotic stimuli and development' category were also found to be differentially regulated in P665 comparing to Messire. Up-regulated proteins included ripening-related proteins, cold- and wound-inducible proteins. An auxin-induced protein and a putative 16.9 kDa heat shock protein were down-regulated.

### qRT-PCR

In general, M values obtained by qRT-PCR showed the same trend as those obtained by microarray (Table 2). However, the sequence MT014356, showing similarities with 12-oxophytodienoate reductase (OPR2), was up regulated according to the microarray experiment but down regulated according to qRT-PCR. In addition, the gene 6a-hydroxymaackiain methyltransferase showed also almost no regulation in the qRT-PCR experiment while was down regulated in the microarray experiment.

**Table 2:** qRT-PCR validation of 10 differentially expressed genes according to microarray experiment

**Oligo ID**^**a**^	**TIGR ID**^**b**^	Annotation	**Hai**^**c**^	**M**^**d **^**microarray**	**M qRT-PCR**^**e**^
MT014704	TC80412	Peroxidase	48	1.56	2.99
MT006316	TC82368	Disease resistance response protein 39 precursor	16	1.29	0.91
MT014356	TC85808	12-oxophytodienoate reductase (OPR2)	16	1.27	-1.62
MT014726	TC79559	glutathione S-transferase	16	1.96	2.25
MT007682	TC77455	nine-cis-epoxycarotenoid dioxygenase1	16	1.12	0.21
MT000707	TC86358	6a-hydroxymaackiain methyltransferase	16	-2.13	-0.03
MT000671	TC86307	ferredoxin--NADP+ reductase	48	-1.2	-2.3
MT014197	TC85300	chlorophyll a/b-binding protein	48	-1.34	-1.33
MT007613	TC86304	GA protein	48	-2.28	-2.65
MT014137	BF633423	ribulose 1 5-bisphosphate carboxylase small subunit	48	-0.92	0.32

^a ^Oligo ID, identifier of *M. truncatula *70-mer oligonucleotides

^b ^TIGR ID, identifier in the TIGR *M. truncatula *Gene Index;

^c ^Hai = hours after inoculation with *M. pinodes*

^d ^M = log_2 _expression ratio P665/Messire

^e ^Primers used to amplify the genes are shown in Table 4

Quantitative RT-PCR techniques was used not only to validate the microarray data, but also to investigate the expression level of 10 selected genes in non-inoculated P665 and Messire plants. That allowed a calculation of the fold changes of different genotype and treatment combinations and hence provided interesting extra-information about the regulation of these genes.

The gene *PsOXII*, encoding a peroxidase, was more expressed in P665 than in Messire after inoculation with *M. pinodes *(Table 3). That was mainly due to a constitutively higher expression level of this gene in P665, as *PsOXII *was up-regulated after inoculation in both genotypes but in a similar amount. That was also the case of the disease resistance response protein 39 (DRR230-b), where the higher expression level of the gene in P665 after inoculation was also due to a constitutively higher expression in P665.

**Table 3:** Log_2 _of normalized expression ratios according to qRT-PCR

Gene	PI/MI	PC/MC	PI/PC	MI/MC
Peroxidase (PsOXII)	2.99	3.8	0.83	0.84
Disease resistance response protein 39 (DRR230-b)	0.91	1.30	1.20	1.63
12-oxophytodienoic acid 10,10-reductase (OPR1)	-1.62	-1.22	0.37	3.5
Glutathione S-transferase	2.25	2.11	0.30	0.55
Nine-cis-epoxycarotenoid dioxygenase 4 (nced4)	0.20	0.04	-0.60	-0.89
6a-hydroxymaackiain methyltransferase (hmm6)	-0.03	0.93	1.06	2.41
Ferrodoxin NADP oxidoreductase	-2.3	-0.5	-0.87	1.13
Chlorophyll a/b-binding protein	-1.33	-0.13	-1.4	0.02
GA protein	-2.6	-0.8	-1.6	0.31
Ribulose 1 5-bisphosphate carboxylase small subunit	0.32	0.6	0.07	0.19

Log_2 _of normalized expression ratios of 10 genes in control (C) and inoculated (I) plants of lines P665 (P) and Messire (M) according to qRT-PCR

12-oxophytodienoic acid 10,10-reductase was down-regulated in P665 as compared to Messire after inoculation according to qRT-PCR. That was caused by a high induction of this gene after inoculation in Messire, while the expression of this sequence was almost not induced after inoculation with *M. pinodes *in P665. In addition, this sequence was constitutively less expressed in P665 than in Messire.

A glutathione S-transferase like gene was constitutively around 4 times more expressed in P665 than in Messire and was slightly induced after inoculation in both genotypes. As a result, P665 inoculated plants showed a higher level of expression of this gene than those of Messire.

The *nced4 *gene, encoding a nine-cis-epoxycarotenoid dioxygenase 4, had a similar regulation pattern in both genotypes. Thus, both genotypes possessed a constitutively similar level of expression of the gene and in both genotypes the gene was repressed after inoculation with *M. pinodes*. Consequently, the level of expression of this gene in P665 was similar to that of Messire after inoculation.

*hmm6 *gene, encoding a 6a-hydroxymaackiain methyltransferase, was constitutively around 2 times more expressed in P665 than in Messire. After inoculation with *M. pinodes *this gene was over expressed in both genotypes but more strongly in Messire. As a result this gene showed a similar level of expression in both genotypes after inoculation.

Ferrodoxin NADP oxidoreductase was less expressed in P665 than in Messire after inoculation. This gene was repressed in P665 after inoculation with *M. pinodes *but induced in Messire. In addition, in P665 control plants this gene was less expressed than in Messire ones.

Messire plants inoculated with *M. pinodes *showed a higher level of expression of chlorophyll a/b-binding protein than P665 plants. Constitutively, both genotypes had a similar level of expression of the gene but this protein was repressed after inoculation in P665 while it was not differentially regulated in Messire.

Messire plants inoculated with *M. pinodes *showed also a higher expression level of a GA protein encoding gene than P665. In this case, the gene was also repressed after inoculation in P665 and only slightly induced in Messire. In addition, the level of expression of the gene in control plants was lower in P665 than in Messire.

The gene encoding ribulose 1 5-bisphosphate carboxylase small subunit showed almost no regulation after inoculation with *M. pinodes *in both genotypes and was not differentially expressed in control plants of both genotypes.

## Discussion

Resistance to *M. pinodes *in pea is a complex trait. Only incomplete resistance to this disease has been identified and genetic analyses have shown that numerous genomic regions are involved in resistance [13-17]. In addition, the necrotrophic nature of *M. pinodes *complicates the performance of histological studies to elucidate the mechanisms of resistance acting to this pathogen. As a consequence, very little is known about the genes and mechanisms of resistance conferring resistance to this important disease. The present study offers a global view of genes and metabolic pathways expressed in a resistant interaction with *M. pinodes *and hence provides an excellent tool to increase our knowledge about pea-*M. pinodes *interaction and to identify candidate genes useful for marker assisted selection.

Previous studies have given some insight into defence responses induced after infection with *M. pinodes *or treatment with a *M. pinodes *elicitor. These studies were performed in susceptible pea lines and showed that the *M. pinodes *elicitor induced the production of the phytoalexin pisatin, the enzymes chalcone sintase and phenylalanine ammonia-lyase, PR proteins as chitinase and endo-b-1,3-glucanase and the generation of superoxide anion. ATPase activity and polyphosphoinositide metabolisms were also activated. On the other hand, *M. pinodes *produces two suppressors that inhibit these defence responses [23-28]. The present study is the first report on genes differentially expressed after infection with *M. pinodes *in a resistant line. We compared gene expression profiling in this resistant line with that of a susceptible line using the microarray technology. This approach can contribute to the identification of the specific genes and mechanisms conferring resistance to *M. pinodes *in pea.

The microarray technology allows the simultaneous assessment of the expression of thousands of genes, being an excellent tool to characterize, at the transcription level, several processes such as defence response to pathogens. As sequence information in pea is limited, we used a microarray containing 70-mer oligos representing all tentative consensus sequences (TCs) of the TIGR *M. truncatula *Gene Index 5. We obtained a successful cross-hybridization between pea targets and *M. truncatula *probes. That result was expected due to the high level of homology and syntheny between these two species [29-31]. A high level of data quality and reproducibility was achieved through the use of tree independent biological replicates and two technical replicates, the use of negative controls and a strict statistical analysis to select the genes differentially expressed.

Comparison between microarray and qRT-PCR results showed common expression kinetics for many of the genes indicating that this microarray experiment is a useful tool to select candidates genes potentially involved in resistance to *M. pinodes *in pea. However, our results also indicate that the involvement of these candidate genes in resistance to *M. pinodes *must be verified by qRT-PCR using *Pisum *sequences. Differences observed between microarray and qRT-PCR may be due to the presence of different gene isoforms or to the cross-hybridization between *M. truncatula *probes and different *Pisum *genes having similar sequences. For example, microarray experiment showed that a *M. truncatula *sequence showing similarities to the gene 12-oxophytodienoate reductase (OPR2) was more expressed in P665 than in Mesire. OPR genes are highly similar to each other. Therefore, to investigate by qRT-PCR which OPR gene was up regulated we used a primer pair based on the sequence of the *Pisum *gene OPR1. But these primers were also able to amplify the genes OPR2, OPR3, OPR4 and OPR6. So, is possible that we have amplified by qRT-PCR sequences corresponding to different genes belonging to this family showing different expression levels. Therefore, further experiments using primers specific for each OPR gene are needed to discern which of them is more expressed in P665 than in Messire after inoculation with *M. pinodes*. In the case of the gene '*hmm6*, 6a-hydroxymaackiain methyltransferase,'in the microarray there were a *M. truncatula *sequence (MT000707) showing similarities to this gene that was strongly down regulated in P665 comparing to Messire. However, when primers were designed according to the pea sequence of this gene (NCBI accession U69554.1) and the gene was amplified by qRT-PCR, results obtained showed that this gene was not differentially regulated in P665 comparing to Messire. Therefore it is possible that other pea genes, different from '6a-hydroxymaackiain methyltransferase' but having a sequence similar to the probe MT000707, or other unknown isoforms of the gene are also able to hybridise to this probe interfering in the results. In addition, the probe MT000707 has homology with the fragment of the gene located from 1041 to 1094 pb (accession U69554.1), while for qRT-PCR we used a pair of primers that amplified the region of the gene located between 730 and 809 pb. Therefore, as we have checked different fragments by microarray and qRT-PCR, another possibility is that P665 differs from Messire in the fragment of the gene corresponding to the probe MT000707. That hypothesis could be further clarified by amplifying by qRT-PCR the fragment of the gene located between 1041 and 1094 pb.

Plants express a wide range of defence responses that can contribute to resistance to pathogens. These include preformed structural and chemical components, activation of the phytoalexin biosynthetic pathway, production of PR proteins, cell wall reinforcement mediated by hydrogen peroxide and detoxification of fungal toxins. Our results suggest that several of these mechanisms may contribute to resistance to *M. pinodes *in pea accession P665.

In interactions with susceptible genotypes, *M. pinodes *spores germinate producing a germ tube and penetrate the pea cuticle directly through the wall of the epidermal cells. Beneath the cuticle, hyphae grow within the outer wall of the epidermis being predominantly aligned with the longitudinal axis of the epidermal cells. Subsequently hyphae grow further within the periplasmic space between plasmalemma and wall, displacing the cell contents, but not causing collapse of the protoplast. This probably biotrophic phase is followed by a necrotrophic one in which *M. pinodes *causes necrotic lesions in the pea mesophyll. These necrotic lesions rapidly spread in susceptible genotypes and hyphae can grow beyond the necrotic zone [32,33]. Previous histological studies performed by the authors [34] indicated that in P665 a proportion of *M. pinodes *infection units were stopped at the epidermal cells not being able to reach the mesophyl. This lower success in establishing colonies was associated with a rapid death of the epidermal cell that was being infected, resembling a hypersensitive response. In addition, those infection units that succeeded penetrating the epidermis and reached the mesophyll in P665 resulted in lesions significantly smaller than those formed in the susceptible line Messire. These results suggest that a battery of resistance mechanisms are acting in P665, starting from a barrier stopping the infection of *M. pinodes *at the epidermis and further barriers acting after the pathogen has penetrated epidermis and reached the mesophyll restricting the growth of *M. pinodes *in the mesophyll. Several genes involved in cell wall fortification were found to be more expressed in P665 than in Messire in the microarray experiment. The involvement of wall reinforcement in the resistance to *M. pinodes *in pea has been suggested by Clulow and Lewis [33] and Wroth [12]. This cell wall reinforcement could contribute to the development of physical barriers hampering the expansion of *M. pinodes *within the P665 tissues or reducing the diffusion of pathogenic toxins. In our microarray experiment 'repetitive proline-rich cell wall proteins', that are structural proteins of the primary cell wall involved in cell wall strengthening, and a 'caffeic acid O-methyltransferase' involved in the lignin synthesis were up regulated in P665 comparing to Messire. In addition, enzymes involved in the production of H_2_O_2_, such as 'peroxidases' and 'amine oxidase' were found also to be up-regulated. H_2_O_2 _is thought to be required for lignification of the cell wall and for the oxidative crosslinking of hydroxyproline-rich glycoproteins in the cell wall [35,36]. The accumulation of reactive oxygen species (ROS) is also associated with the occurrence of hypersensitive response [37] that may play a role in the resistance of line P665 to *M. pinodes*. ROS can also be toxic and inhibit fungal growth [38] and act as signaling agents in plant defense [39].

In addition to ROS, other compounds have antimicrobial properties and can contribute to the inhibition of pathogens development. The recognition of a pathogen by the plant activates several defensive responses including the activation of the phenylpropanoid metabolism and the production of phytoalexins [40]. Phenylpropanoids are a group of plant secondary metabolites derived from phenylalanine that have a wide variety of functions, including defense against microbial attacks or other sources of injury. Phytoalexins are plant antibiotics that are synthesized after the plant tissue is exposed to microbial infection. The production of these antifungal compounds has a relevant role in plant defence. Our results indicate that many enzymes involved in their synthesis were higher expressed in P665 than in Messire after inoculation with *M. pinodes*. Those included 'UDP-glycose flavonoid glycosiltransferase', 'coumarate CoA ligase like protein', 'phenylalanine ammonia-lyase', 'flavanone 3 beta-hydroxylase' and 'hyoscyamine 6 beta-hydroxylase'. However, other enzymes of these pathways as 'flavonol synthase', 'tropinone reductase" and "amygdalin hydrolase isoform AH I precursor" were down regulated.

In addition, qRT-PCR data showed that the enzyme '6a-hydroxymaackiain methyltransferase', which catalyses the last step of the synthesis of pisatin, the main pea phytoalexin, is constitutively at a higher concentration in P665 than in the susceptible cultivar Messire. This enzyme was activated after inoculation with *M. pinodes *in both genotypes and both genotypes showed similar amount of this enzyme 16hai. The constitutively higher expression of this enzyme in P665, suggests that in P665 pisatin can start acting earlier against the pathogen and can reach the same final level as Messire with a lower effort by the plant.

Pathogenesis- related (PR) proteins are also induced during infection by pathogens and several of them possess antimicrobial properties [41]. The PR14 are 'lipid transfer protein' and a sequence encoding such a protein was up regulated in P665. Another up-regulated PR protein encoded a precursor of the defensin 'disease resistance response 39 (DRR230-b)'. This gene was induced after infection with *M. pinodes *and showed a constitutively higher expression in P665 comparing to Messire. DRR230-b defensin was first identified by Chian and Hadwiger [42] from pea pods in response to infection by the fungal pathogen *Fusarium solani*. The gene encoding 'disease resistance response 39 precursor' was also present in a cDNA library obtained from a resistant *Lathyrus sativus *accession inoculated with *M. pinodes *[43]. More recently, this defensin was found to co-localize with the QTL *mpIII-4 *involved in field resistance to *M. pinodes *in pea [18]. In addition, the related defensins DRR230-a and DRR230-c were also found to be induced after infection with several pathogens including *A. pinodes *(the teleomorph of *M. pinodes*) [44]. Our results reinforce these recent studies suggesting the important role of this protein in resistance to diseases in pea, and specially in resistance to *M. pinodes*.

Necrotrophic fungi, as *M. pinodes*, kill host tissues during infection, usually through the secretion of toxic substances. Therefore, the ability of a plant to detoxify these fungal toxins may contribute to resistance to necrotic pathogens. Thus, chickpea cultivars with higher sensitivity to the phytoxins produced by *Ascochyta rabiei *are more susceptible to this pathogen [45]. Our results show that P665 may posses a higher ability to detoxify *M. pinodes *toxins as two genes involved in detoxification processes, the 'glutatione S-transferase' and 'ABC transporter', were found to be more expressed in P665 than on Messire. Glutatione S-transferases are involved in several metabolic processes and in the detoxification of a wide variety of compounds including microbial toxins [46]. ATP-binding cassette transporters (ABC-transporter) are transmembrane proteins that function in the transport of a wide variety of substrates across extra- and intracellular membranes including toxins, drugs, glutatione conjugates, peptides and secondary metabolites [47,48].

In addition to the genes reported above, other genes involved in defence were also up-regulated in P665. These included a "syringolide-induced protein" that have been found to be induced after treatment with the syringolide elicitors produced by the bacteria *Pseudomonas syringae *[49]. Other up-regulated genes with a possible involvement in defence were genes encoding lectins, as several plant lectins have been shown to induce the production of pisatin [50].

Perception of both general and specific pathogen associated molecules triggers defence responses via signal transduction cascades and transcriptional activation of numerous genes [51]. The expression of transcription factors and proteins kinases, as well as elevation of cytosolic calcium, is integral to the signalling of these defences [52]. We identified several genes involved in signal recognition and transduction pathways, such as kinases, CCCH-type zinc finger protein and transcription factors, that were differently expressed in P665 comparing to Messire after inoculation with *M. pinodes*. Among the differentially regulated transcriptional factors there were some associated with Jasmonic Acid (JA) and Ethylene (ET): "ethylene responsive element binding factor-like", "transcription factor JERF1" and "pathogenesis related transcriptional factor ERF". This suggests that the response to *M. pinodes *in pea is regulated via JA and ET pathways. This is in agreement with the predominant necrotrophic nature of *M. pinodes*, as gene-for gene resistance and SA signalling are generally effective against biotrophic pathogens whereas JA/ET signalling is generally effective against necrotrophs [53].

In addition to genes involved in defence against pathogens also genes involved in response to abiotic stresses and development such as 'ripening-related protein-like', 'CIC protein cold-inducible' and 'wound-induced protein T9A4.6' were more expressed in P665 than in Messire showing that response to abiotic and biotic stresses and proteins involved in development are interlinked, as many other studies suggest. For example a "ripening related protein" was found also to be expressed in a pea line resistant to *Erysiphe pisi *[54] and in the model legume legume *Medicago truncatula *in response to the parasitic plant *Orobanche crenata *[55].

Our results suggest that resistance to *M. pinodes *in P665 is in part due to a constitutively higher expression of genes involved in defense such as peroxidases, DRR230-b, GST and 6a-hydroxymaackiain methyltransferase. The first step in the response to a pathogen is the recognition of the pathogen by the plant. This recognition leads to the induction of the defence responses. In gene-for-gene resistance, early recognition of specific pathogen strains, a key step in a successfully defense, depends on complementary pairs of dominant genes, one in the host and one in the pathogen. Gene-for-gene resistance is common in interactions with many biotrophic pathogens [56]. In contrast, resistance mediated by a single host resistance gene is uncommon in the case of necrotrophic fungal pathogens. In the case of necrotrophic pathogens plants usually recognize non-specific elicitors that activate a battery of basal defense responses that act against a wide range of pathogens. In this case, as is the case of resistance to the necrotrophic fungi *M. pinodes*, the recognition of a pathogen is not so fast and a preformed higher expression of genes with antimicrobial properties can be an advantage to get a fast and effective defence response.

## Conclusions

In this study, we have obtained a global view of genes expressed during resistance to *M. pinodes*. This gave us information about the possible mechanisms and pathways involved in the resistance to this important disease such as cell wall reinforcement, production of phytoalexins, phenylpropanoids and PR proteins and detoxification of fungal toxins. This study is also an useful tool to identify candidates genes involved in the control of resistance to *M. pinodes *in pea useful for marker assisted selection. Further studies will include the mapping of the most relevant genes identified in this study in a RIL population derived from the cross P665 x Messire where QTLs associated with resistance to *M. pinodes *have been identified and functional analysis to discern the role of these genes in resistance.

## Methods

### Plant material and inoculation

Two pea genotypes, P665 and Messire, the parental lines of a RIL population previously used to identify QTLs associated with resistance to *M. pinodes *[13] were used in the experiment. Messire is a commercial *Pisum sativum *ssp. *sativum *cultivar highly susceptible to *M. pinodes*. P665 is a *P. sativum *ssp. *syriacum *accession displaying incomplete resistance to *M. pinodes *[11]. Previous histological studies revealed that resistance to *M. pinodes *in accession P665 was characterized by a lower succeed in colony establishment, associated with the rapid death of the epidermal cell being attacked by *M. pinodes *and by a smaller colony size [34].

For inoculation plants were grown until the fifth leave stage in a growth chamber (20 ± 2°C with a 12 h dark/12 h light photoperiod, at 250 μmol m^-2 ^sec^-1^). Plants were inoculated with the monoconidial *M. pinodes *isolate C0-99, obtained from infected pea material collected in commercial fields at Córdoba, Spain. The isolate was multiplied in Petri dishes containing V8 juice medium located in a growth chamber at 21 ± 2°C with a 12 h dark/12 h light photoperiod, at 106 lmol/m^2 ^s. A spore suspension was prepared by flooding the surface of 12 days old cultures with sterile water, scraping the colony with a needle and filtering the suspension through two layers of sterile cheesecloth. The concentration of spores in the solution obtained was further determined with a haemocytometer and adjusted to 350.000 spores per ml. Finally, Tween-20 (120 μl per 100 ml of suspension) was added as a wetting agent and the spore suspension was applied with a sprayer at a rate of 1 ml per plant. After inoculation high humidity was ensured during the first 24 h by ultrasonic humidifiers operating for 15 minutes every two hours. After that period the humidifiers were turned off.

The experiment was performed in three independent replicates, each having 3 to 5 plants per genotype (Messire/P665), treatment (inoculated/control) and time of harvesting (16, 24 and 48 hours after inoculation). In each replicate, plants grown under the same conditions but not inoculated were used as control.

#### Sample collection and RNA extraction

At 16, 24 and 48 hours after inoculation (hai) leaflets of control and inoculated plants were harvested, immediately frozen in liquid nitrogen and stored at -80°C. RNA was isolated using Trizol reagent (Invitrogen, Karlsruhe, Germany) according to manufacture's protocols. Integrity of total RNA was checked on agarose gels and its quantity, as well as purity, was determined using NanoDrop ND1000 (NanoDrop Technologies, Inc., Wilmington, USA). RNA from infected plants was further purified and concentrated to 0.8 μg/μl using Microcon-30 YM columns (Millipore, Schwalbach, Germany).

#### Microarray experiment

Microarray experiment was performed at the Institute for Genome Research of Bielefeld University, Germany. For each time of harvesting and replicate, Cy-labelled cDNA samples from resistant and susceptible inoculated plants labelled with different Cy dies were co-hybridized to Mt16kOLI1Plus microarray as described by Küster et al. [57]. The experiment included three biological and two technical replicates incorporating one dye swap. The resulting images were analysed using the ImaGene 5.5 software (Bio-Discovery, Los Angeles) as described by Hohnjec *et al*., [58]. Data files were imported into the EMMA1.1 array analysis software [59] and normalized using Lowess normalization. To identify the genes differentially expressed in the inoculated resistant genotype compared to the susceptible one a t-test followed by FDR correction was performed. Genes were considered differentially regulated when p ≤ 0.05 and M ≤ -0.8 or M ≥ 0.8, being M = Log_2 _(red/green). The microarray data have been deposited into the public data base ArrayExpress (E-TABM-1084).

#### Data validation by quantitative real time Reverse Transcription PCR (qRT-PCR)

The expression profiles of 10 genes differentially expressed according to the microarray experiment were validated in inoculated and control plants using two steps qRT-PCR. Total RNA was extracted from different samples obtained from the same three replicates used for microarray study using TRISure (Bioline, London, UK). After checking its quality, any possible residual genomic DNA was removed using RQ1 RNase-Free Dnase (Promega, Madison, USA). RNA was further purified using RNeasy Plant Mini Kit (Quiagen, Hilden, Germany). The absence of genomic DNA was checked by PCR using specific primers that amplify and intron-exon-intron sequence of the *P. sativum *gene glyceraldehyde-3-phosphate dehydrogenase (GAPDH) (Fw: 5'-3': GTGGTCTCCACTGACTTTATTGGT/Rv 5'-3': TTCCTGCCTTGGCATCAAA, Die et al., 2010). Total RNA (5 μg) was reverse-transcribed using SuperScript III First-Strand Synthesis System for RT-PCR (Invitrogen, Karlsruhe, Germany).

In order to ensure equal starting cDNA amounts, real-time PCR amplification of α-Tubuline (TUB) was run for all the different templates and, depending on the CT (threshold cycle) number, cDNA samples were diluted to obtain similar CT values. In addition, to check the quality of the reverse transcription, specific primers were used to amplify in each template two fragments of the gene GAPDH located 915 bp apart at the 5'or 3'end of the transcript (GAPDH1 Fw 5'-3': ctccactgactttattggtgaca/Rv 5'-3': caaacttgtcatttaaggcaattc; GAPDH2 Fw 5'-3': tcaagatcggaatcaacggatt/Rv 5'-3': cgagttcaacatcatctctcttcaa).

Polymerase chain reactions were performed in a 96-well plate with a 7500 Real Time PCR System (Applied Biosystems, Foster City, CA, USA), using SYBR Green to monitor dsDNA synthesis. Reactions contained 0.5 μl of Fast Start Universal SYBR Green Master (ROX), 1 μl of cDNA, and 0.3 μM of each gene-specific primer in a final volume of 10 μl. The following standard thermal profile was used for all PCR reactions: polymerase activation (95°C for 10 min), amplification and quantification cycles repeated 40 times (95°C for 15 seconds, 60°C for 1 min) and dissociation curve (95°C 15 seconds, 60°C 1 min, 95°C 30 seconds).

*P. sativum *sequences with similarities to 5 *M. truncatula *microarray probes up-regulated in P665 compared to Messire and 5 down regulated were retrieved from NCBI data Base and used to design gene-specific primers using Probe Finder 2.45 (Universal Probe Library, Roche). The genes validated and primer sequences used are shown in Table 4. In order to cover the range of variation of times points studied in the microarray experiment, the validation of the expression profiles of the genes by qRT-PCR was done with samples obtained at 16hai for five genes and with samples obtained at 48 hai for other 5 genes.

**Table 4:** Primers used to amplify 10 candidate genes by qRT-PCR

Gene	Forward primer (5'-3')	Reverse primer (5'-3')	Reference accession
Peroxidase (PsOXII)	cttggaggacccacatggat	tttggcttgctgttcttgca	GenBank:AB193816.1
Disease resistance response protein 39 (DRR230-b)	gggagtatgcttcacgaatgc	ttttgagtgcagaaacatttcca	GenBank: LO1579.1
12-oxophytodienoic acid 10,10-reductase (OPR1)	aagtgaatgacagaaccgatga	atggaaaccgacagcgatt	GenBank: AY954368.1
Glutathione S-transferase	gttcgtcctcctccgctaact	gttcgtcctcctccgctaact	GenBank: AB087837
Nine-cis-epoxycarotenoid dioxygenase 4 (nced4)	ctctcttcccgaacgtcttctc	cgcacgtggatccataaccgcc	GenBank: U69554.1
6a-hydroxymaackiain methyltransferase (hmm6)	tttgaactttgttggtggagatatg	aatcatgcagaacccacttgagt	GenBank: U69554.1
Ferrodoxin NADP oxidoreductase	acaagcaagtgtgccgaaagt	ggaggttcagaaaggattttcca	GenBank: X99419.1
Chlorophyll a/b-binding protein	gttttcgcatcaacggactt	attgcccaccagggtaaag	GenBank: EF488077.1
GA protein	tgcagacagctttaacctttgc	tgcgagacactctttggtgttg	GenBank: X65154.1
Ribulose 1 5-bisphosphate carboxylase small subunit	caagtcttgaaggagcttgatgaa	gttgtcgaaaccgatgatacga	GenBank: J01257.1

The genes TUB, histone H3 and GAPDH [60] were used as reference genes for normalization.

The PCR efficiency of each primer pair in each individual reaction was calculated using LingRegPCR 7.5 software and used to calculate an average efficiency (E) per primer pair. This average efficiency was used to calculate the expression in each reaction using the formula Expresion = E^CT^. A normalization index was calculated for each plate as the geometric mean of the expression of the reference genes TUB, GAPDH and histone H3 and a relative expression was calculated for each reaction as the ratio of the gene expression of the gene of interest in each reaction against the normalization index.

## Authors' contributions

SF carried out the inoculations with *M. pinodes*, extracted the RNA, performed the qRT-PCR assays and drafted the manuscript. HG performed the microarray hibridizations. FK was involved in the microarray experiment design and data analysis and in the critical revision of the manuscript. JIC carried out a critical revision of the manuscript. DR conceived the study, provided the funding to do the experiments and supervised the research. All authors read and approved the final manuscript.

## Supplementary Material

Additional file 1 **Table S1**. Genes differentially expressed in the resistant accession P655 comparing to the susceptible one cv Messire at 16, 24 and 48 hours after inoculation (hai) with *M. pinodes*. Genes induced are listed according to the functional categories as defined by Journet et al. (2002) and are sorted within these classes according to the induction level at 16hai. Oligo ID = identifier of *M. truncatula *70 mer oligonucleotides. F.C = functional categories as defined by Journet et al. (2002). M = log2 (expresion ratio). TIGR ID = identifier in the TIGR *M. truncatula *Gene Index. Annotation = annotations according to TIGR release http://compbio.dfci.harvard.edu/tgi/ Empty cells means that no significance was detected at P < 0.05 or M > -0.8 or M < 0.8. Click here for file
